# Effect Investigation of Process Parameters on 3D Printed Composites Tensile Performance Boosted by Attention Mechanism-Enhanced Multi-Modal Convolutional Neural Networks

**DOI:** 10.3390/polym18020203

**Published:** 2026-01-12

**Authors:** Zeyuan Gao, Zhibin Han, Yaoming Fu, Huiyang Lv, Meng Li, Xin Zhao, Jianjian Zhu

**Affiliations:** 1 College of Aviation Engineering, Civil Aviation Flight University of China, Chengdu 641400, China; gaozeyuan@cafuc.edu.cn (Z.G.); ymfo@163.com (Y.F.); lvhuiyang@cafuc.edu.cn (H.L.); limeng@cafuc.edu.cn (M.L.); 2 School of Aerospace Engineering, Xiamen University, Xiamen 361005, China; zhibinhan@xmu.edu.cn

**Keywords:** 3D printing, process parameters, attention mechanism, multi-modal learning convolutional neural network

## Abstract

Fused Deposition Modeling (FDM) is a widely used additive manufacturing technique that enables the fabrication of components using polymeric and composite materials; however, the mechanical performance of printed parts is jointly influenced by multiple printing parameters, which complicates the control and prediction of their mechanical properties. In this study, an attention-enhanced multi-modal convolutional neural network (ATT-MM-CNN) is developed to predict the tensile performance of carbon fiber reinforced polylactic acid (PLA-CF) composites manufactured by FDM. Four key printing parameters, layer thickness, nozzle temperature, material flow rate, and printing speed, are systematically investigated, resulting in 256 parameter combinations and corresponding tensile test data for constructing a multi-modal dataset. By integrating multi-modal feature representations and incorporating an attention mechanism, the proposed model effectively learns the nonlinear relationships between printing parameters and mechanical performance under multi-parameter conditions. The results show that all evaluation metrics, including accuracy, precision, recall, and F1-score, exceed 0.95, and the prediction accuracy is improved by at least 17.3% compared with baseline models. These findings demonstrate that the proposed ATT-MM-CNN provides an effective and reliable framework for tensile property prediction and process-parameter optimization of FDM-printed composite structures.

## 1. Introduction

Additive Manufacturing (AM), also known as 3D printing, is a cutting-edge manufacturing process that is fundamentally distinct from traditional subtractive processes [1]. It enables the fabrication of complex geometric structures without the need for molds or tooling, thereby offering high design flexibility. By depositing materials in a layer-by-layer manner, AM significantly improves material utilization efficiency and reduces manufacturing costs. Owing to these advantages, 3D printing has rapidly advanced in recent years and has been widely applied in aerospace, mechanical engineering, and medical industries [2]. Among various 3D printing technologies, Fused Deposition Modeling (FDM) has attracted particular attention due to its simplicity, low cost, and material versatility [3]. During the FDM process, thermoplastic composites are heated to a molten state and extruded through a nozzle along a predefined path, followed by layer-by-layer deposition to form three-dimensional solid structures [4]. In FDM applications, the mechanical properties of printed components serve as critical indicators of print quality and are influenced by multiple factors, including material type, printing parameters, and environmental conditions. However, quantitatively characterizing the influence of these factors—particularly printing parameters—on mechanical performance remains a core challenge in this field [5].

To enhance the mechanical performance of FDM-fabricated components, extensive research efforts have focused on both material selection and process optimization [6]. Regarding material development, commonly used thermoplastics such as polylactic acid (PLA) [7], polyethylene terephthalate glycol (PETG) [8], and acrylonitrile butadiene styrene (ABS) [9] offer advantages including ease of processing, cost-effectiveness, and, in some cases, biodegradability. Nevertheless, the mechanical properties of unreinforced thermoplastics are fundamentally governed by polymer chain deformation and intermolecular interactions within the polymer matrix, which provide limited resistance to external stress compared with rigid reinforcement phases. Moreover, the viscoelastic behavior of thermoplastics gives rise to time- and temperature-dependent deformation, such as creep and stress relaxation, leading to progressive degradation of stiffness and strength under sustained loading and demanding service conditions. Consequently, it remains challenging for pure thermoplastics to satisfy the mechanical reliability and durability requirements of high-load applications. To address these intrinsic material limitations, increasing attention has been directed toward fiber-reinforced thermoplastic composites. Systems such as PLA-CF/GF and ABS-CF/GF have demonstrated notable improvements in strength, stiffness, and thermal stability through fiber reinforcement, showing strong potential for aerospace and other load-critical applications [10,11,12]. The development of these composite materials provides a solid material basis for further enhancing the mechanical performance of FDM-printed structures.

Alongside material advances, the influence of printing parameters on the mechanical properties of FDM-printed components has been extensively investigated [13]. Previous studies have demonstrated that individual parameters, such as printing speed, layer thickness, nozzle temperature, and material flow rate, exert significant effects on structural strength [14,15]. Most existing investigations adopt a control-variable approach, in which one parameter is varied while others are held constant to evaluate its individual effect. Although this approach is straightforward, it inherently overlooks the interaction effects arising from the simultaneous variation in multiple printing parameters, such as the combined influence of thermal-related parameters (e.g., nozzle temperature and printing speed) and geometrical or deposition-related parameters (e.g., layer thickness, infill configuration, and build orientation), thereby limiting its ability to represent practical printing conditions [16]. In addition, physics-based modeling approaches have been employed to analyze the tensile behavior of FDM specimens, primarily focusing on thermal and mechanical phenomena during the printing process. For example, Alarifi [17] investigated the combined effects of multiple printing parameters on PETG performance through numerical simulations and experiments, while Khanafer et al. [18] developed a three-dimensional model to predict interlayer bonding and optimize parameters using Design of Experiments (DOE). Although these methods are effective in specific cases, they are often computationally intensive and rely on simplifying assumptions, which limit their flexibility for practical parameter optimization.

To improve the understanding and optimization of FDM process–property relationships, researchers have explored a variety of analytical and modeling approaches to examine the influence of multiple printing parameters [19]. Among these efforts, systematic statistical techniques such as orthogonal experimental design, response surface methodology (RSM), and the Taguchi method have been widely adopted, as they enable the analysis of multiple parameters using relatively small experimental datasets and facilitate the identification of dominant effects and limited interactions. For example, El Magri and Vaudreuil [20] applied RSM to optimize printing temperature, speed, and layer thickness for PLA–graphene composites, while Maidin et al. [21], Kam et al. [22], and Cicek and Johnson [23] employed Taguchi-based and ANOVA-assisted methods to explore multi-parameter effects in ABS, PA12, and polycarbonate systems. Although these approaches are effective when the number of considered parameters is limited, their applicability gradually becomes restricted as the parameter space expands and interactions become more complex [15]. In response to these challenges, data-driven methodologies have attracted increasing attention, and machine learning (ML) has emerged as a powerful alternative for performance prediction and process analysis [24]. In the context of FDM, various ML algorithms, including artificial neural networks (ANNs), decision tree-based models, and convolutional neural networks (CNNs), have been employed to establish relationships between printing parameters and mechanical properties [24,25,26,27,28], providing an effective means of capturing complex parameter dependencies in FDM-fabricated composite structures.

However, conventional ML approaches are typically designed to learn from a single form of input representation, which is insufficient for fully characterizing the complex mapping between printing parameters and mechanical performance. In this study, the same set of printing parameters can be represented from different perspectives, and relying on only one representation may result in incomplete feature learning [29]. To address this limitation and enable more effective feature extraction and integration, the present study proposes an attention-enhanced multi-modal convolutional neural network (ATT-MM-CNN). The experimental design considers four critical printing parameters—nozzle temperature, layer thickness, printing speed, and material flow rate—each examined at four levels, resulting in 256 unique parameter combinations. Standard tensile tests were conducted to measure fracture strength and construct a labeled dataset. Structured process parameters and printed part images were treated as one-dimensional (1D) and two-dimensional (2D) modalities, respectively. Features from each modality were independently extracted through dedicated convolutional branches and subsequently fused using a multi-modal integration strategy. An attention mechanism was incorporated to enhance feature representation by emphasizing the most informative components.

The proposed ATT-MM-CNN model was evaluated using four standard performance metrics: accuracy, precision, recall, and F1-score. The results demonstrate the effectiveness of the proposed framework in handling heterogeneous and high-dimensional data. By leveraging multi-modal learning, the model captures latent correlations across different feature types, while the attention mechanism further improves its ability to focus on task-relevant information. Consequently, this approach provides a robust methodological pathway for optimizing FDM printing parameters and predicting the mechanical performance of composite structures.

Finally, the remainder of this paper is organized as follows. Section 2 presents the theoretical background and architectural design of the ATT-MM-CNN. Section 3 describes the experimental setup, data processing, and dataset construction. Section 4 discusses and analyzes the results in detail, and Section 5 concludes the study and outlines directions for future research.

## 2. Materials and Methods

### 2.1. Principle of FDM-Based 3D Printing

**Figure 1:**
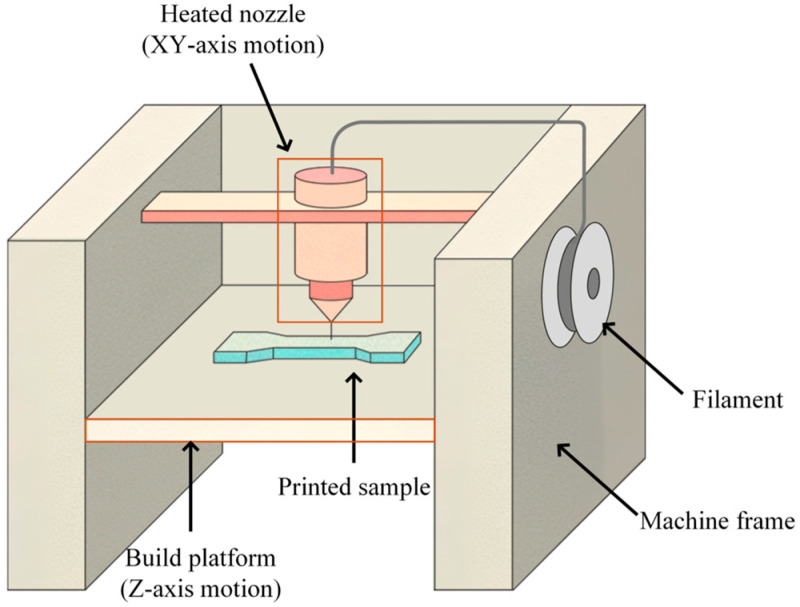
Schematic illustration of the Fused Deposition Modeling printing process.

This study adopts the FDM technique, which is a core process in AM and operates through the melting, deposition, and sequential solidification of thermoplastic materials [30]. The FDM workflow can be divided into three main stages: 3D modeling, model slicing, and layer-by-layer fabrication. The process begins with the creation of a digital prototype using computer-aided modeling software. The completed model is then imported into dedicated slicing software, which converts the 3D geometry into a series of two-dimensional (2D) toolpath instructions. During printing, a filament feeding system continuously delivers the thermoplastic filament to a heating element, where it is melted and extruded through a nozzle (typically 0.1–0.4 mm in diameter) integrated with the heater. The molten material is deposited sequentially along a predefined trajectory onto the build platform, forming each layer successively. After cooling and solidification, strong interlayer adhesion is established, and continuous stacking along the *Z*-axis ultimately results in a fully formed 3D structure, as illustrated in Figure 1.

### 2.2. Fundamentals of Multi-Modal CNN

Convolution is a fundamental mathematical operation that is extensively applied in signal and image processing, and is typically performed in 1D or 2D forms. Building upon this principle, the present study proposes a multi-modal learning framework that integrates both 1D and 2D data representations to enhance predictive accuracy [31].

CNN is a class of deep learning architecture that is particularly effective for processing grid-structured data, such as images. CNNs are inspired by biological visual systems and are capable of automatically learning hierarchical feature representations from input data [32]. They have been widely applied to tasks including image classification, object detection, and semantic segmentation, and have also been extended to domains such as natural language processing and speech recognition. In a typical CNN architecture, local features are extracted through convolutional layers, spatial dimensionality is reduced, and overfitting is mitigated via pooling layers, and classification or regression is performed using fully connected layers. Moreover, the weight-sharing mechanism employed in CNNs significantly reduces the number of trainable parameters, thereby improving computational efficiency and generalization performance, particularly in scenarios with limited training data.

**Figure 2:**
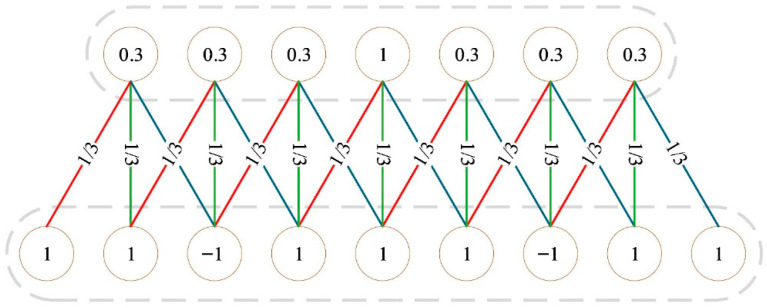
Schematic of 1D convolution operation.

Its mathematical formulation is expressed in Equation (1):

(1) $$y(t)=(x\times w)(t)=\sum_{i=0}^{M-1}x(t-i)w(i)$$

where x(t) represents the input signal, w(i) denotes the convolution kernel, y(t) is the output signal after convolution, and M is the length of the convolution kernel. Figure 2 illustrates the process of extracting low-frequency and high-frequency features using different filters.

**Figure 3:**
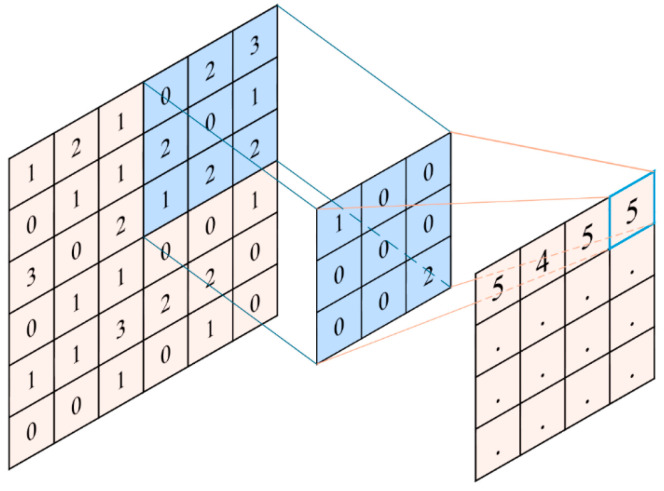
Schematic of a 2D convolution operation.

2D convolution is extensively applied in image processing, where a convolution kernel traverses local regions to extract spatial features. Its formulation is expressed as Equation (2):

(2) $$O(x,y)=(I\times K)(x,y)=\sum_{m=0}^{M-1}\sum_{n=0}^{N-1}I(x-m,y-n)K(m,n)$$

where I is the input image, K is the convolution kernel, and O(x,y) is the output feature map. Figure 3 demonstrates image smoothing achieved through mean filtering. Convolution operations effectively capture local features from input data, facilitating deeper learning in subsequent layers.

Compared with traditional approaches, CNN utilizes automatic feature learning and parameter-sharing mechanisms to effectively capture complex relationships between printing parameters and mechanical properties, thereby enabling the development of high-accuracy predictive models.

To further enhance predictive performance, this study incorporates a multi-modal learning strategy. Multi-modal learning refers to the integration of information from heterogeneous data sources to enhance the comprehension and predictive capability of models [33]. Common fusion strategies include early fusion, mid-level fusion, and late fusion [34]. Early fusion integrates data from multiple modalities at the input level to generate a unified feature vector, as described in Equation (3):

(3) $$F_{fused}=[F_{1};F_{2};\ldots ;F_{N}]$$

where $X_{i}$ is the input feature of the *i*-th modality, and $F_{fused}$ is the fused feature vector.

Late fusion, on the other hand, involves performing feature extraction and model training independently for each modality, followed by the fusion of their outputs after training. A common late fusion strategy is weighted averaging, formulated as Equation (4):

(4) $$y_{final}=\sum_{i=1}^{N}\omega_{i}\cdot y_{i}$$

where $y_{i}$ is the output of the *i*-th modality, $\omega_{i}$ is its corresponding weight, and $y_{final}$ is the fused output. Mid-level fusion integrates features from different modalities at intermediate layers of the model, often employing neural network structures such as attention mechanisms or gating mechanisms to optimize and fuse features.

This study adopts a late fusion approach, where features extracted from each modality are combined after independent processing, and the fused features are then fed into a subsequent classifier or regression model. A key challenge in late fusion is determining how to integrate features from multiple modalities into a unified representation to support decision-making.

Suppose there are N different modalities, with the feature of each modality represented as $f_{i}\in R^{d_{i}}$ and is the feature dimension of the *i*-th modality. In late fusion, common fusion methods include concatenation, weighted summation, and voting. The fused feature $f_{fusion}$ can be obtained through concatenation, Equation (5):

(5) $$f_{fusion}=Concatenate(f_{1},f_{2},\ldots ,f_{N})$$

or via weighted summation, Equation (6):

(6) $$f_{fusion}=\sum_{i=1}^{N}w_{i}\cdot f_{i}$$

where $w_{i}$ is the weight assigned to the i-th modality, typically determined through learning or manual tuning. Concatenation links the features of each modality into a single long vector, while weighted summation computes a weighted average of the features from different modalities.

**Figure 4:**
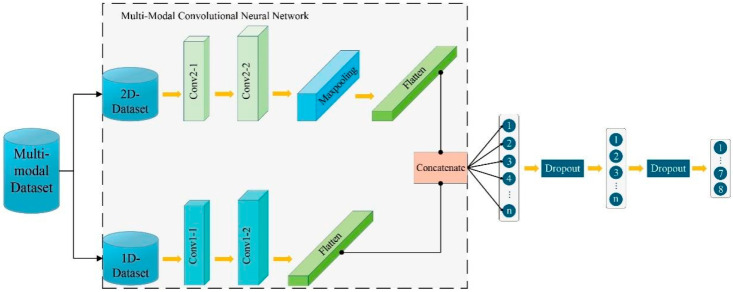
Architecture of the proposed MM-CNN.

In this study, the 1D and 2D data streams are processed independently through separate network branches, where features are extracted and subsequently fused to perform classification tasks. The model operates under a collaborative optimization framework designed for task-specific objectives such as classification, regression, or detection. This multi-modal fusion approach enables the network to acquire a more holistic understanding of complex, multi-factorial problems, thereby enhancing both its performance and generalization capabilities. The baseline architecture of the proposed Multi-Modal Convolutional Neural Network (MM-CNN), prior to the incorporation of any attention mechanisms, is depicted in Figure 4. This architecture provides the structural foundation for the attention-enhanced model introduced in the following section.

### 2.3. Principle of Channel Attention Mechanism

The channel attention mechanism, which has been widely adopted in recent years, offers significant advantages in multiple aspects. This mechanism dynamically adjusts the weights of individual channels, effectively emphasizing features critical to the task at hand, thereby substantially enhancing model performance. Additionally, it improves the interpretability of the model, rendering its behavior more transparent and traceable. Furthermore, the channel attention mechanism strengthens the generalization capability of the network, enabling it to better adapt to diverse and unseen data distributions [35]. These benefits have accelerated the widespread adoption of channel attention mechanisms in deep learning and computer vision applications, and their advantages are particularly relevant to this study. In the context of our collected multi-modal dataset, the channel attention mechanism enables the model to selectively emphasize the most task-relevant features within each modality while capturing the complex interdependencies induced by multi-parameter coupling, thereby enhancing predictive performance.

The principle of the channel attention mechanism lies in dynamically recalibrating the feature map by focusing on the importance of different feature channels [36]. Specifically, this mechanism computes weights for each channel and applies these weights to the feature map, thereby amplifying or suppressing the feature responses of different channels. These weights are learned automatically during the training process. The core idea involves initially compressing the feature maps of each channel using global average pooling as in Equation (7) or global max pooling in Equation (8), which aggregates spatial information into a global descriptor. This process can be expressed as follows:

(7) $$Z_{c}=\frac{1}{H\times W}\sum_{i=1}^{H}\sum_{j=1}^{W}X_{i,j,c}$$

(8) $$Z_{c}=\underset{i,j}{\max}\;X(i,j,c)$$

where H and W represent the height and width of the feature map, C denotes the number of channels, and $Z_{c}$ is the pooling value for the C channel. Subsequently, fully connected layers are employed to capture inter-channel dependencies and generate weight coefficients, as expressed in Equation (9) and detailed in Equation (10):

(9) $$Z=[z_{1},z_{2},\ldots ,z_{c}]$$

(10) $${s=\sigma (W}_{2}\times {ReLU(W}_{1}\cdot z))$$

where $W_{1}$ and $W_{2}$ are the weight matrices of two fully connected layers, r is the reduction ratio, $ReLU\left(\cdot \right)$ is the ReLU activation function, σ(·) is the Sigmoid activation function, and *s* is the generated channel weight vector [37]. Finally, these weight coefficients are multiplied by the original feature map to recalibrate the channel responses, as shown in Equation (11):

(11) $$X_{i,j,c}^{\prime }=s_{c}\times X_{i,j,c}$$

**Figure 5:**
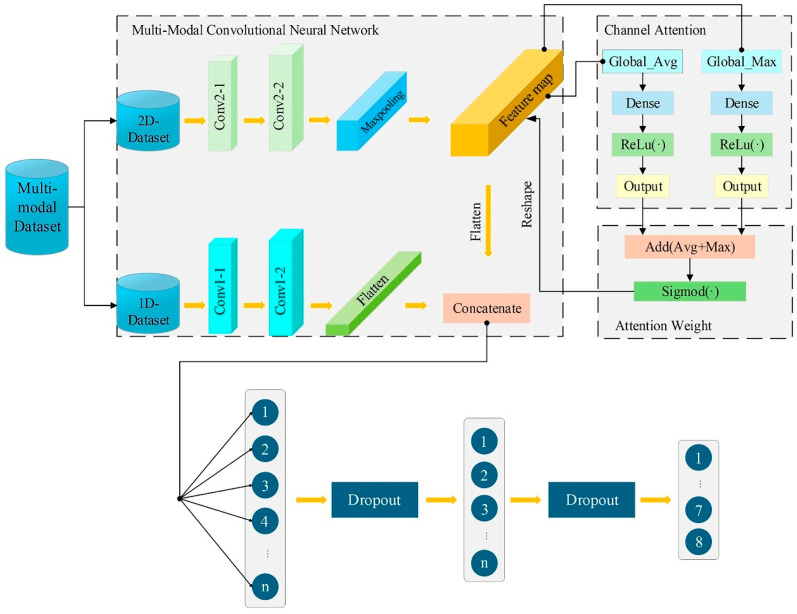
Architecture of the proposed ATT-MM-CNN.

Channel attention enables the network to selectively focus on task-relevant feature channels, enhancing overall performance. To improve feature representation and fusion capability, a channel attention mechanism is integrated into the 2D processing branch of the MM-CNN. Specifically, as shown in Figure 5, the network incorporates a channel attention module after the second convolutional layer in the 2D branch. This module utilizes both global max pooling and global average pooling to capture the feature maps from different aspects, followed by fully connected layers that generate channel-wise attention weights. These weights are then applied to the feature maps, allowing the network to emphasize the most informative features before fusion with the 1D feature representations. This attention-driven enhancement contributes to more effective multi-modal integration and improves classification accuracy.

## 3. Experimental Configuration and Dataset Construction

### 3.1. Raw Materials and Fabricating Platform

**Figure 6:**
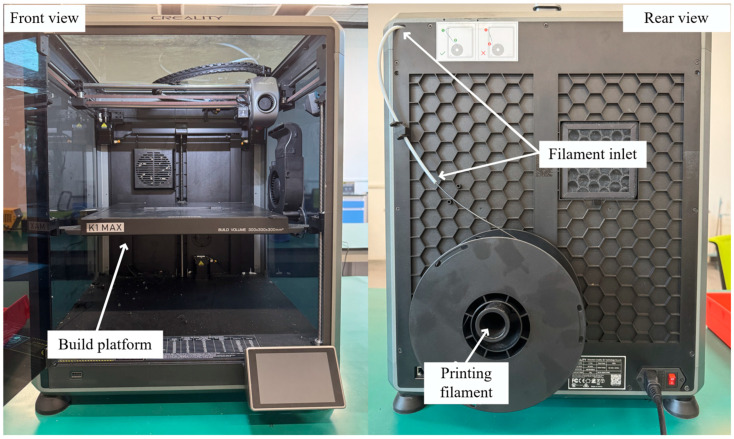
Illustration of experimental platform and materials.

The experiments in this study utilized a PLA-CF composite filament designed for FDM processing(DOWELL 3D, Shenzhen, China). The filament had a diameter of 1.75 mm and incorporated 12 wt.% short carbon fibers. During printing, the filament spool was mounted at the rear of the FDM printer(Creality K1 Max, Creality, Shenzhen, China), as illustrated in Figure 6. This composite combines the biodegradability of PLA with the high strength of carbon fiber, offering new opportunities for industrial 3D printing applications. Owing to the biodegradability of PLA and the reinforcement from carbon fibers, the composite provides improved strength and stiffness, making it suitable for industrial additive manufacturing applications.

### 3.2. Sample Fabrication and Testing

**Figure 7:**
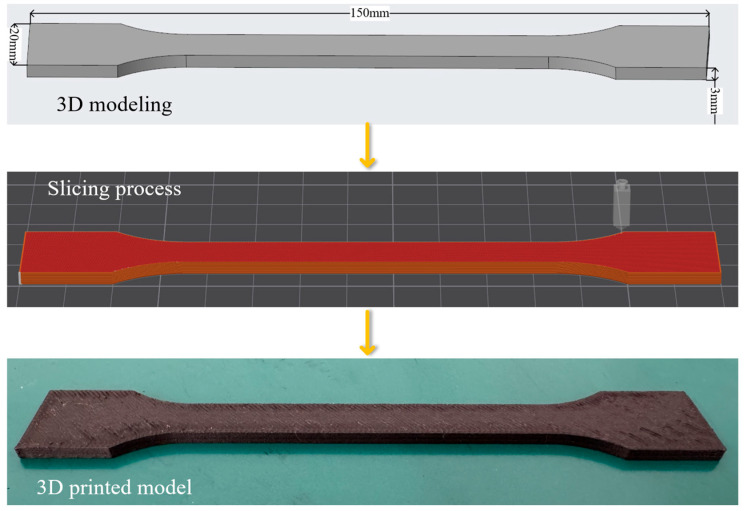
Procedure of FDM-printed samples for tensile testing.

As discussed previously, the tensile behavior of FDM-printed specimens is highly sensitive to the configuration of process parameters. In this study, four representative parameters—layer thickness, printing speed, material flow, and nozzle temperature—were selected to examine their influence on the tensile response. The specific parameter values are summarized in Table 1. Each factor was assigned four levels, and a full-factorial design was adopted to generate 256 printing conditions, allowing a systematic evaluation of multi-parameter interactions. The tensile specimen geometry was defined according to the dimensional requirements of the Chinese National Standard GB/T 1040.2-2006 [38] (Plastics—Determination of Tensile Properties—Part 2: Test Conditions for Molding and Extrusion Plastics), based on the parameter settings in Table 1, and fabricated by FDM printing. Representative printed samples are shown in Figure 7.

**Figure 8:**
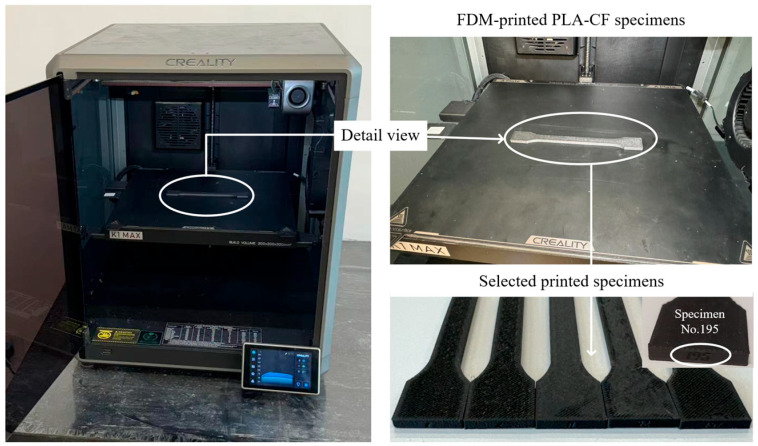
FDM printing system deployed in experiments.

The tensile specimens had dimensions of 150 mm × 20 mm × 3 mm, as shown in Figure 7. After modeling, the files were imported into proprietary slicing software, where printing parameters were configured, models were sliced, and each experimental specimen was assigned a unique identifier, yielding 256 specimens. Figure 8 illustrates the FDM fabrication workflow and the layer-by-layer construction of the specimens produced in this study.

**Figure 9:**
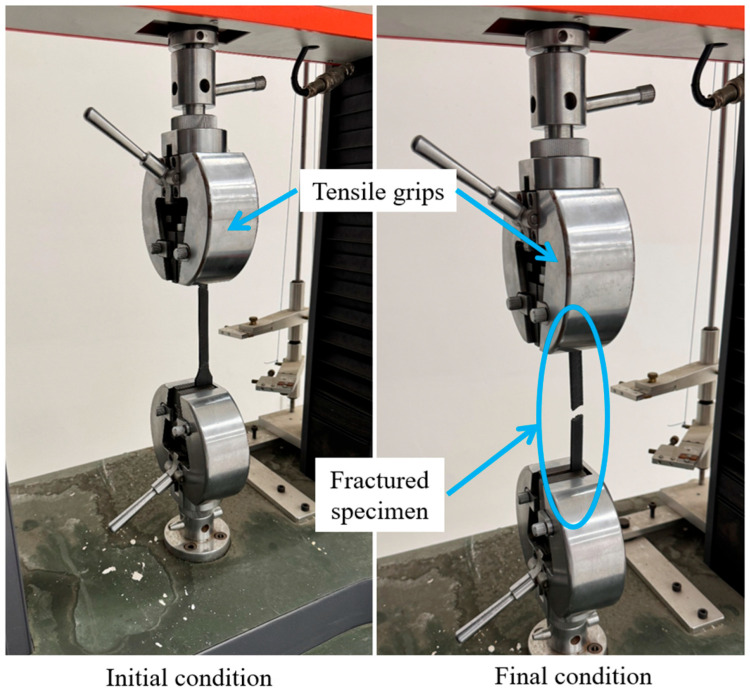
Tensile strength testing for FDM-printed samples.

Upon completion of the printing process, tensile tests were performed using a universal tensile testing machine in accordance with the Chinese National Standard GB/T 13729-2010 [39] (Test Method for Tensile Properties of Carbon Fiber Reinforced Plastic Materials), as shown in Figure 9. Precise data were acquired via a computer interfaced with the tensile testing machine. To improve the reliability of the tensile measurements, the testing system was checked and zeroed before each run, and specimens were carefully aligned in the grips to reduce eccentric loading. All specimens were tested using the same loading protocol and data acquisition settings, and the specimen width and thickness were measured prior to testing and used for stress calculation. These practices help control the main contributors to experimental uncertainty and ensure consistent comparability across the different printing conditions.

### 3.3. Dataset Construction Based on 3D-Printed Samples

**Figure 10:**
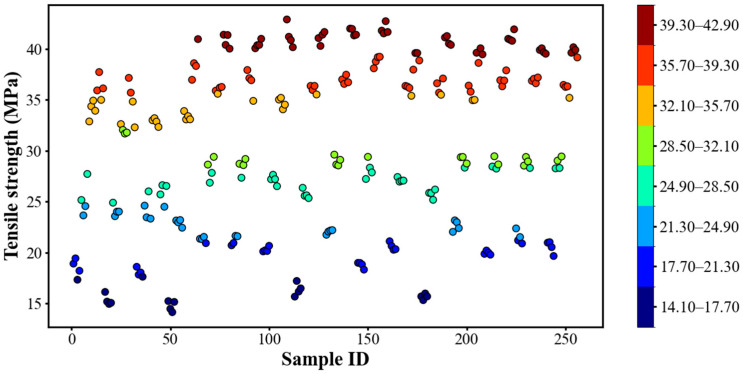
Tensile strength distribution of FDM-printed samples.

The tensile strength values obtained from the experiments ranged from 14.13 MPa to 42.90 MPa. Based on this distribution, the data were divided into eight classes (C01–C08) using an interval of 3.60 MPa. Table 2 lists the tensile strength range corresponding to each class. Figure 10 presents the strength distribution of the 256 PLA-CF specimens, where each color-coded point denotes a distinct combination of printing parameters, highlighting the variability in tensile performance induced by different parameter configurations.

**Figure 11:**
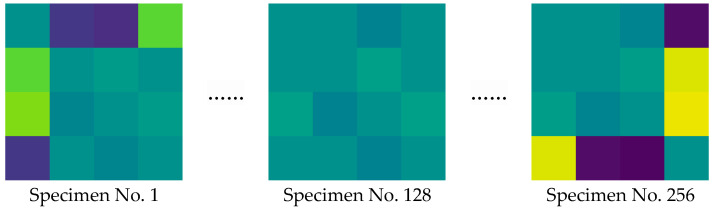
Transformed GAF images based on the original dataset.

Meanwhile, due to the low dimensionality of the original data, directly using the raw numerical parameters may be insufficient to capture complex interrelationships among them. To address this issue, the Gramian Angular Field (GAF) method is employed to map the 1D parameters to a polar coordinate system, thereby generating structured 2D image features. Since it is impractical to display all GAF-transformed images in a single paper, a subset is shown for illustration in Figure 11.

After data grouping, the Synthetic Minority Oversampling Technique (SMOTE) was applied to augment the data and expand the sample size. The dataset was then split into a training set (80%, used for model training and optimization) and a testing set (20%, used for independent evaluation of generalization). These images clearly exhibit distinct visual patterns after transformation. This approach facilitates pattern recognition, feature extraction, and visualization, enabling the construction of a multi-modal dataset that integrates 1D numerical data with 2D image representations, and then the related data are fed into the MM-CNN for training, respectively.

## 4. Results and Discussion

### 4.1. Influence of Printing Parameters on Tensile Performance

**Figure 12:**
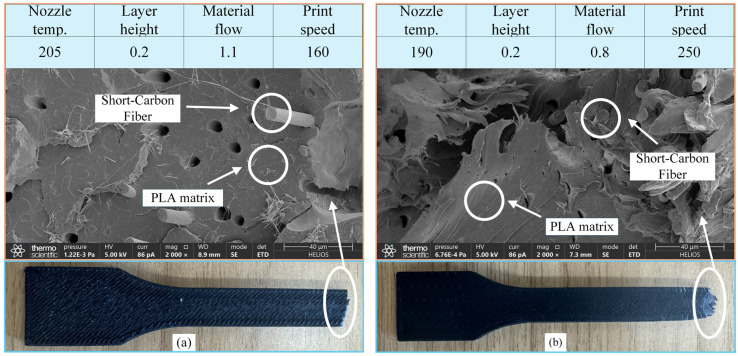
Comparison of fracture morphology under different FDM printing parameters: (**a**) Flattening fracture surface, (**b**) Tearing fracture surface.

To elucidate the effects of printing parameters on the fracture mechanisms of PLA-CF specimens, the fracture surfaces were examined using scanning electron microscopy. Figure 12 presents representative morphologies under two typical parameter settings, with the distributions of Short Carbon Fibers (SCF) and PLA matrix annotated for clarity.

Under the printing condition of 205 °C nozzle temperature, 0.2 mm layer thickness, 110% flow rate, and 160 mm·s^−1^ printing speed (Figure 12a), the fracture surface exhibits a smooth and compact morphology with well-developed interlayer fusion. SCF is partially embedded within the PLA matrix, forming a comparatively integrated fiber–matrix interface. This morphology suggests that adequate thermal input and sufficient material supply promote melt wetting and polymer chain diffusion across adjacent layers, thereby enhancing the resultant tensile performance. In contrast, the specimen fabricated under the less favorable parameter combination (Figure 12b) manifests pronounced tearing trajectories and interlayer delamination, accompanied by a noticeably rougher fracture surface. The reduced nozzle temperature together with the elevated printing speed suppresses melt flowability and hinders interlayer consolidation, giving rise to weak interfacial adhesion and, consequently, inferior mechanical strength.

These comparative observations show that the coupling effects of nozzle temperature, material flow rate, printing speed, and layer height significantly influence the tensile behavior of FDM-printed composites. Optimizing these parameters is essential for enhancing structural cohesion and overall mechanical performance. Based on the analysis of these two cases, different combinations of printing parameters have a pronounced impact on the fracture behavior and mechanical performance of the samples. This confirms that multi-parameter optimization is essential for improving the structural reliability of FDM-printed components.

After analyzing different fracture morphologies, this section will conduct a unified analysis of the printing parameters for each group to observe the influence of printing parameters on tensile properties. The dataset was categorized into four groups based on different nozzle temperatures (190 °C, 205 °C, 220 °C, and 235 °C), and the results are visualized in the 3D scatter plot in

**Figure 13:**
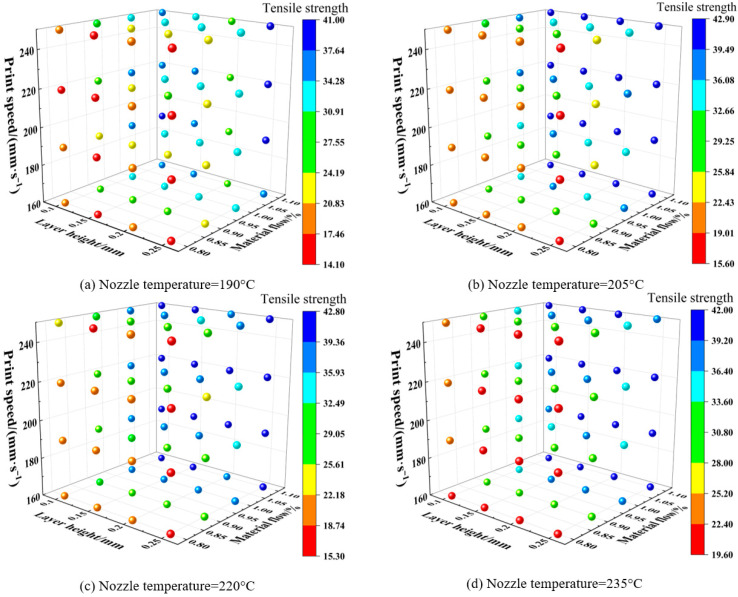
Schematic of 3D scatter of the FDM-printed samples.

Figure 13 presents 3D scatter plots for each fixed nozzle temperature based on the control variable method. The color bar at right encodes tensile strength from low to high, and the points in each plot represent specific printing-parameter sets. Each plot contains 64 data points, yielding 256 total parameter sets. The data show that the tensile strength of the samples at a nozzle temperature of 205 °C has a high value and a concentrated distribution; at 190 °C, the tensile strength is lower and the dispersion is larger, indicating that the PLA-CF does not melt sufficiently at this temperature and exhibits poor interlayer bonding; at a nozzle temperature of 220 °C, the thermal degradation of the material leads to a polarized strength distribution, where some samples maintain high strength but the overall stability decreases; this trend is further aggravated at 235 °C, confirming the degradation threshold of temperature on material properties.

Figure 13 enables a more intuitive assessment of how parameter combinations influence strength. These data help construct a more complete dataset and serve as a reference for optimizing 3D-printing parameters in future composite work (e.g., PLA-CF). While the image effectively visualizes the relationship between tensile strength and printing parameters, the mutual influence among various printing parameters remains complex. To address this complexity, the ATT-MM-CNN architecture is introduced to learn the nonlinear mapping between printing parameters and tensile performance, enabling accurate prediction under multi-parameter coupling.

### 4.2. Impact of Hyperparameters on ATT-MM-CNN

This study constructed a dataset using the classification criteria in Table 2 and applied SMOTE to address class imbalance across groups, thereby supporting the training of an ATT-MM-CNN. Numerical features were processed through a 1D convolutional model, whereas image features were analyzed using a 2D convolutional model.

**Figure 14:**
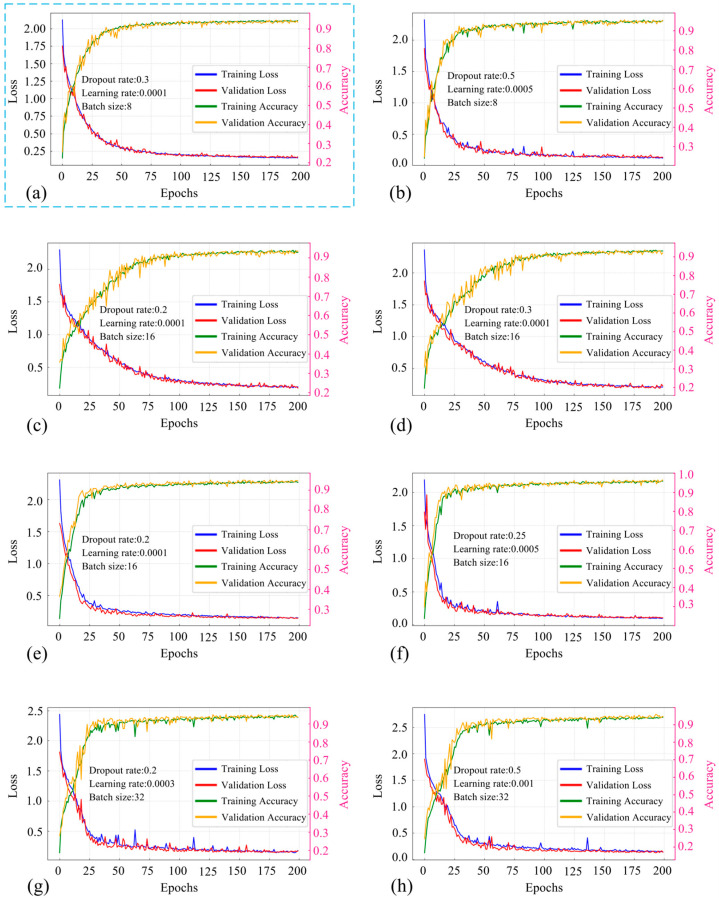
Diagram of performance comparison for different hyperparameters from (**a**) OHP; (**b**–**h**): HC1-HC7.

To further improve the performance of the proposed ATT-MM-CNN, Bayesian hyperparameter optimization was employed, targeting three critical hyperparameters, which are dropout rate, learning rate, and batch size. The impact of different Hyperparameter Combinations (HC) on the fitting performance of the model is systematically investigated, as illustrated in Figure 14. Detailed hyperparameter settings are provided in Table 3, which lists representative configurations for training the ATT-MM-CNN. As shown in Figure 14, the training and testing results demonstrate that different HCs affect model fitting performance, as reflected by the accuracy and loss curves. Among these configurations, the optimal hyperparameter combination obtained through Bayesian optimization is shown in Figure 14a and is highlighted with a blue bounding box, indicating the best overall fitting performance in terms of accuracy and loss.

**Figure 15:**
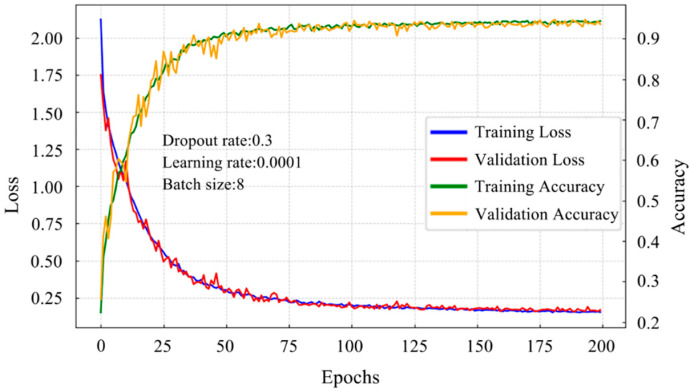
Fitting the history of the ATT-MM-CNN using the dataset and optimized hyperparameters.

The combined loss and accuracy curves presented in Figure 15 depict the training history of the ATT-MM-CNN under the optimal hyperparameter configuration. The curves show a well-aligned performance trend between the training and validation sets throughout the entire learning process. During the early stage of training, specifically within the first 25 epochs, the accuracy increases rapidly while the loss decreases sharply, indicating fast convergence and effective feature learning. At this stage, the network demonstrates strong adaptability and generalization ability, achieving accuracy levels above 90% while substantially reducing the loss. Beyond 25 epochs, the accuracy curves of the training and validation sets remain highly consistent, and the loss gradually stabilizes around 0.1 with only minor fluctuations. This stable behavior reflects robust generalization and the absence of overfitting. The sustained agreement between the two curves further confirms that the model maintains reliable learning dynamics as training progresses. Moreover, High accuracy and persistently low loss beyond approximately 175 epochs further highlight the robustness of the network and its ability to jointly exploit one-dimensional numerical features and two-dimensional image-based representations. These results also verify the effectiveness of the multi-modal fusion mechanism in enhancing feature representation and predictive performance.

Overall, the results verify that the joint learning of 1D numerical features and 2D image-based representations through a multi-modal fusion strategy is effective. The architecture successfully captures complex cross-modal relationships and delivers strong classification performance, accompanied by a stable and well-behaved training process.

### 4.3. Influence Assessment of Printing Parameters on Tensile Performance

#### 4.3.1. Validating Model Performance from Multiple Perspectives

In this study, four metrics—accuracy, precision, recall, and F1-score—were systematically employed to evaluate the model, with the corresponding scores detailed in Table 4. The experimental results show that the model achieves an accuracy of 0.9650 on the testing set, demonstrating strong predictive capability in correctly classifying diverse samples. A precision score of 0.9518 further highlights the reliability of the model in identifying positive-class samples while minimizing false positives. Additionally, the recall score of 0.9650 underscores the strong ability of the model to detect the majority of true positive cases. The F1-score, defined as the harmonic mean of precision and recall, reaches 0.9650, indicating a well-balanced trade-off between precision and recall.

The combined performance across all four metrics clearly demonstrates the superiority of the proposed model. Notably, it maintains high precision to ensure accurate predictions, while simultaneously achieving high accuracy and recall, thereby contributing to stable and reliable overall performance. This balanced performance highlights the model’s strong practical potential for real-world applications.

**Figure 16:**
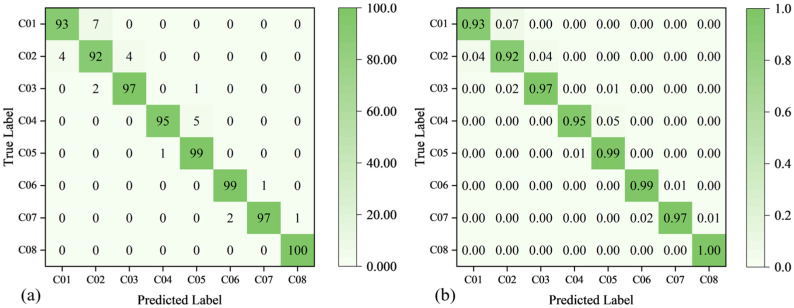
CM diagrams of tensile strength prediction using the dataset: (**a**) non-normalized CM, (**b**) normalized CM.

The Confusion Matrix (CM) is a critical tool for evaluating the performance of deep learning models, particularly in multi-class classification tasks. To visualize the prediction performance of the model on the test set, this study presents the CM of the ATT-MM-CNN, as shown in Figure 16.

Figure 16 shows two CM plots labeled (a) and (b), which illustrate the performance of a classification model across eight categories, ranging from C01 to C08. Figure 16a presents the raw sample counts for true versus predicted labels. The diagonal entries represent the number of correctly classified samples for each category, such as 100 samples for C01, whereas off-diagonal entries indicate misclassifications, like 7 samples of C01 being misclassified as C02. Figure 16b conveys classification accuracy and error rates. Diagonal values denote the accuracy for each class, with C08 achieving 100% accuracy (1.00) and C01 achieving 93% accuracy (0.93). Off-diagonal values represent the proportion of misclassified samples, exemplified by 7% of C01 samples being misclassified as C02 (0.07). These CM plots collectively reveal that the model performs well, with most errors occurring between adjacent categories. Overall, Figure 16a provides detailed counts of correct and incorrect classifications, while Figure 16b offers a clearer perspective on the accuracy of ATT-MM-CNN and error distribution, facilitating a comprehensive evaluation of its performance.

In terms of tensile strength classification, Figure 16 further demonstrates that the proposed ATT-MM-CNN model performs consistently well, achieving classification accuracies exceeding 95% in six out of eight categories. Even for the relatively less accurately predicted categories, namely C01 and C02, the model maintains accuracy levels above 92%. Overall, these results confirm that the ATT-MM-CNN model is highly effective and reliable for evaluating tensile performance.

Collectively, the evaluation metrics and CM analysis demonstrate the strong classification capability of the proposed ATT-MM-CNN model. The model achieves consistently high accuracy across the majority of categories, thereby establishing a robust and reliable framework for practical deployment in real-world applications. This level of performance further highlights the model’s considerable potential for advancing process–property optimization in 3D-printed composite materials.

#### 4.3.2. Comparison Between ATT-MM-CNN and Classic Methods

To comprehensively evaluate the performance of the proposed ATT-MM-CNN, a comparative analysis was conducted against several traditional ML models. The experimental dataset was used independently to train each model, and four standard evaluation metrics—accuracy, precision, recall, and F1-score—were recorded after the training process.

**Figure 17:**
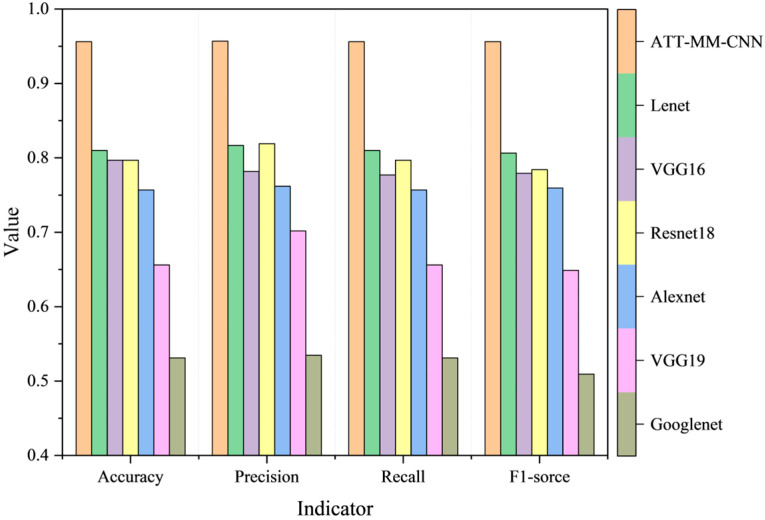
Comparison between the proposed ATT-MM-CNN and classic methods.

As illustrated in Figure 17, the comparative results demonstrate that the ATT-MM-CNN consistently achieves higher scores across all evaluation metrics than the conventional ML approaches. In the bar chart, the height of each bar corresponds to the numerical value of the respective metric, where taller bars indicate better performance, thereby providing a clear and intuitive comparison of the overall advantage of the proposed framework.

A direct comparison between the ATT-MM-CNN and the traditional CNN, as shown in Figure 17, further confirms the superior predictive capability of the proposed network under identical dataset configurations. Overall, the results indicate that the ATT-MM-CNN delivers high predictive accuracy and strong robustness, offering a reliable and practical framework with strong potential for deployment in related engineering applications.

## 5. Conclusions

In this study, an attention-enhanced multi-modal convolutional neural network (ATT-MM-CNN) was developed to predict the tensile properties of FDM-fabricated composite specimens by modeling the complex relationships between printing parameters and mechanical response. The main conclusions can be summarized as follows:

(1) An ATT-MM-CNN framework was successfully constructed to integrate multi-modal feature representations and effectively capture the nonlinear relationships between FDM printing parameters and the tensile performance of composite materials.
(2) The application of the SMOTE algorithm alleviated class imbalance in the grouped dataset, while Bayesian optimization further improved hyperparameter selection, leading to enhanced model stability and overall predictive performance.
(3) Compared with conventional CNN architectures, the proposed model achieved superior and stable performance, with accuracy, precision, recall, and F1-score consistently exceeding 95% on both testing and validation datasets, demonstrating its effectiveness and reliability for tensile property prediction and process-parameter optimization in FDM-printed composites.

Despite these achievements, the present study is limited to a single composite system and does not account for environmental influences. Future work will extend the proposed framework to additional material systems and incorporate environmental factors, such as hygrothermal aging, to further evaluate their effects on mechanical performance.

## Figures and Tables

**Table 1:** Printing parameters of the FDM process used in experiments.

Nozzle Temp	Layer Height	Material Flow	Printing Speed
(°C)	(mm)	(%)	(mm/s)
190	0.1	80	160
205	0.15	90	190
220	0.2	100	220
235	0.25	110	250

**Table 2:** Categories based on fracture stress.

Category	C1	C2	C3	C4	C5	C6	C7	C8
fracture	[14.10,	[17.70,	[21.30,	[24.90,	[28.50,	[32.10,	[35.70,	[39.30,
Stress	17.70)	21.30)	24.90)	28.50)	32.10)	35.70)	39.30)	42.90)

**Table 3:** Hyperparameters to evaluate the influence on ATT-MM-CNN performance.

Items	Space	OHP	HC1	HC2	HC3	HC4	HC5	HC6	HC7
DR	[0.2, 0.5]	0.3	0.35	0.2	0.3	0.4	0.25	0.2	0.5
LR	[10^−4^, 10^−3^]	0.0001	0.0005	0.0001	0.0001	0.0008	0.0005	0.0003	0.001
Batch	[8, 16, 32]	8	8	16	16	16	16	32	32

**Table 4:** Indicator scores for model performance evaluation.

Indicators	Accuracy	Precision	Recall	F1-Score
Scores	0.9650	0.9518	0.9650	0.9650

## Data Availability

The original contributions presented in this study are included in the article. Further inquiries can be directed to the corresponding authors.

## Abbreviations

The following abbreviations are used in this manuscript:

AM	Additive Manufacturing
FDM	Fused Deposition Modeling
PLA	Polylactic Acid
CF	Carbon Fiber
SCF	Short Carbon Fibers
ML	Machine Learning
CNN	Convolutional Neural Network
ATT-MM-CNN	Attention-enhanced Multi-Modal Convolutional Neural Network
RSM	Response Surface Methodology
SMOTE	Synthetic Minority Oversampling Technique
GAF	Gramian Angular Field
SEM	Scanning Electron Microscopy
